# Jamaica Ginger

**Published:** 1891-10

**Authors:** 


Jamaica Ginger contains more alcohol than the strongest whiskey, and aggravates its inflammatory effects with an additional and violent irritant. It is almost unequaled as a cause of uncontrollable inebriety, and should be banished from the house and from public sale.—*Sanitary Era.*

